# Using Decision Trees to Support Classifiers’ Decision-Making about Activity Limitation of Cerebral Palsy Footballers

**DOI:** 10.3390/ijerph18084320

**Published:** 2021-04-19

**Authors:** José M. Sarabia, Alba Roldan, Matías Henríquez, Raul Reina

**Affiliations:** 1 Sport Research Centre, Department of Sports Sciences, Miguel Hernández University, 03202 Elche, Spain; jsarabia@umh.es (J.M.S.); matias.henriquez@alu.umh.es (M.H.); rreina@umh.es (R.R.); 2 Alicante Institute for Health and Biomedical Research (ISABIAL Foundation), 03010 Alicante, Spain

**Keywords:** para-sport, disability, brain impairment, Paralympic, para-football

## Abstract

This study aimed (1) to determine the appropriateness of using decision trees as a classification tool for determining the allocation of sport classes of para-footballers with “moderate vs. mild” cerebral palsy (CP) profiles of spastic diplegia/hemiplegia and ataxia/athetosis based on observational outcomes by international classifiers, and (2) to identify what key observational features were relevant to discriminating among different impairment levels. A sample of 16 experienced international classifiers from five world regions participated in this study, observing activity limitation of a final sample of 21 international CP footballers when performing 16 gross-motor and sports-specific tests for balance (*n* = 3), coordination (*n* = 5), running, accelerations and decelerations (*n* = 3), jumping (*n* = 4), and change of direction ability (*n* = 1). For the overall sample (336 observations), the model included eight decision nodes and 24 branches with 17 leaves, including side-step, side-stepping, and triple hop as the tests with the best sensitivity (precision = 67.0%). For those with spastic diplegia (64 observations: Two nodes, six branches with five leaves), the range of motion in the side-step test and the balance in the tandem walk tests correctly classified 89.1% of the observations. In those with athetosis and ataxia (96 observations), the model included five nodes, 15 branches, and 11 leaves (176 observations, precision = 86.5%). For those with spastic hemiplegia, a model containing two nodes, six branches, and five leaves had 90.9% accuracy, including observational features of balance in the side-step test and symmetry in the side-stepping test. The observational tool used in this study, based on the impact of specific impairment measurements of hypertonia, athetosis, and ataxia, can be used to determine which assessments are more appropriate for discriminating between functional profiles in para-footballers with CP.

## 1. Introduction

Football for people with cerebral palsy (CP), or CP football, is a seven-a-side team para-sport with some differences in the game rules compared with conventional football, such as a reduction in the game time (i.e., two 30 min halves), a smaller field of play (70 × 50 m) and goal (5 × 2 m) size, and the no-offside rule. To compete in CP football, players must have an eligible impairment of hypertonia, ataxia, or athetosis (HAA) when their respective minimum impairment criteria are accomplished [1]. To receive a sport class for competition, CP footballers must participate in a classification process that determines the impact of the neural compromise on the activity limitations related to physical parameters and specific football skills [2]. This classification process aims to provide a framework of eligibility of the participants and to promote their participation by minimising the impact of the impairment on the outcome of the competition [3].

Over three decades, CP football (i.e., since its introduction at the 1984 Paralympic Games until the 2016 Rio Paralympic Games) has used a functional system developed by the Cerebral Palsy International Sports and Recreation Association [4]. The system comprises eight functional classes: The first four groups (classes 1–4) correspond to para-athletes who are wheelchair users, while the last four groups (classes 5–8) host the ambulant para-athletes. These latter para-athletes are eligible for CP football. More specifically, those with “moderate” spastic diplegia, where the function of both legs is affected, are allocated in the sport class FT5; those with a “moderate” ataxic or athetoid profile involving the four limbs and trunk are allocated in the sport class FT6; those with “moderate” spastic hemiplegia, where one side of the body (right/left arm and leg) is affected by spasticity, are allocated in the sport class FT7; and those with “mild” involvement of diplegia, ataxia/athetosis, or hemiplegia, also called the minimum impairment criteria for eligibility in this para-sport, are allocated in the sport class FT8 [5]. This classification structure is also applicable for runners and throwers with CP and related neurological conditions, that is, eligible impairments of HAA for competing in para-athletics [6]. Para-athletes with HAA impairments tend to be the most difficult to classify within the Paralympic Movement. The difficulty lies in determining the severity of the impairment(s) and quantifying its/their impact on sport-specific performance. In CP football, unlike running/jumping or throwing (i.e., closed skills for track and field events in para-athletics), an additional challenge is faced in identifying the relevant components of “performance” as many simultaneous actions are involved (i.e., running, jumping, turning, kicking, changing direction, dribbling, etc.), and weighting them when they take place in a variable environment (i.e., opened skills) [7].

The current classification process in CP football comprises three stages [8]. First, the eligibility and severity of the impairments are based on the application of clinical scales, such as the Modified Ashworth Scale (MAS) [9] for hypertonia, the Scale for the Assessment and Rating of Ataxia (SARA) [10] for ataxia, and the Dyskinesia Impairment Scale (DIS) [11] for athetosis. During this initial physical assessment, balance (i.e., one-leg stance, tandem walk) and coordination (i.e., rapid heel–toe placement, split jumps, side-stepping) are also evaluated. Second, gross-motor movements (i.e., running, change of direction ability, jumping) and specific football skills (i.e., passing, dribbling, kicking, two-a-side game) are evaluated on a football pitch to determine activity limitation. After these two stages, para-athletes receive a provisional sport class that should be confirmed after the observation assessment in the real competition (i.e., the third stage). Valid, reliable, and ratio-scaled measures available to assess the impairment facilitate the quantification of activity limitation [3,12]. However, the classification in team sports should consider some other aspects and open motor skills that significantly impact sports performance and make this process more complex [13].

Evidence-based classification in CP football has already reported several tests to explore determining factors to discriminate between different sport classes and impairment severity (i.e., FT5/FT6/FT7 vs. FT8) and between minimum impairment and controls (i.e., FT8 vs. not eligible), according to abilities such as balance [14], coordination [2,15], horizontal and vertical jumps [16], change of direction [17], sprinting, accelerations and decelerations [18], and dribbling skills [19]. Recently, Reina et al. [2] suggested that the relationship between activity limitation and sports performance should be considered impairment specific for para-athletes with HAA. This statement was based on the variability and specific motor limitation across CP profiles (i.e., spastic diplegia, ataxia/athetosis, and spastic hemiplegia). However, contrary to this, other studies in para-athletics [20,21], para-swimming [22], and RaceRunning [23] have considered a one-dimensional factor of sports performance (i.e., acceleration, sprint time, maximal freestyle swim speed) and categorised the sample of participants with CP as a unitary group (i.e., “coordination impairments”).

Hence, descriptive and statistical procedures to improve sport class allocation are fundamental to the pursuit of an accurate classification process, facilitating standardisation, enhancing objectivity, and ensuring that para-sport actors (i.e., stakeholders, staff, and athletes) understand the classification process [13]. One of the most recent approaches in para-sports to support decision-making class allocation is the application of decision trees. Decision trees are predictive models used to graphically represent and organise information about possible options, consequences, and end values. Karalis [24] argued that decision trees are a way to express the rules underlying data with hierarchical, sequential structures that recursively partition the data. Since they are commonly used for calculating probabilities and help in decision making, this method helps classifiers to assess the relationship between impairment function and para-athlete performance, as it has been already shown in para-shooting [25] and para-judo [26] for athletes with visual impairment. In CP football, data envelopment analysis [15] and cluster analysis [14] have been used as classification methods, while, to the best of the authors’ knowledge, the decision tree has never been applied. While clustering methods oversee grouping sets of data objects intending to find relationships within the objects that compose the data, decision trees allow us to discover the effects of interaction in a specific subgroup of cases previously classified—in sport classes in our case.

After all the above, this study aimed to (1) assess the sensitivity of several activity limitation tests and their ability to discriminate among different levels of impairment in ambulant para-footballers with spastic diplegia, ataxia/athetosis, and spastic hemiplegia, and (2) identify a complementary tool to the existing observation scales to facilitate classifiers’ decision-making for sport classes allocation in CP football players.

## 2. Materials and Methods

### 2.1. Participants

This observational cross-sectional study was conducted with two study samples. On the one hand, a sample of 16 experienced international classifiers (Table 1), with regular classification activity of CP footballers and/or runners with HAA. The representative sample was composed of classifiers from the five regions: Africa (*n* = 3, 18.8%), the Americas (*n* = 4, 25%), Asia (*n* = 2, 12.5%), Australasia (*n* = 4, 25.0%), and Europe (*n* = 3, 18.8%). Eleven classifiers have expertise classifying para-athletes with HAA but their main activity as classifiers is for track and field (i.e., para-athletics) and the remaining eight classifiers were international classifiers for CP football.

On the other hand, a sample of 28 male para-athletes was randomly selected from a larger dataset of the research group for the aims of this study. All were classified as Gross Motor Function Classification System Level I [27]. This sample was from the Americas (four countries, *n* = 15, 71.4%) and Europe (three countries, *n* = 6, 28.6%), and all had international experience participating in world championships and/or Paralympic Games (see the demographics in the Results section since the sample was shortened to 21 players during data reduction). For those that fully completed the test battery (*n* = 60), this sample was selected considering the distribution of CP profiles in the larger data set and their representation in CP football squads (i.e., over-representation of those with unilateral spasticity belonging to the FT7 sport class). Moreover, it should be mentioned that the sample of 28 para-athletes was set considering the above-mentioned reasons and other practical reasons, such as the time required for the classifiers to complete the observations (i.e., from 1.5 to 2 h per player). 

All participants agreed to participate in the study and signed an informed consent form provided before participation. Ethical approval was obtained through the local University Ethics Committee (Ref. DPS.RRV.01.14). 

### 2.2. Materials

An ad hoc observation tool was used in this study following the procedures described by Roldan et al. [28]. The instrument is included in Supplementary Material (File S1) and was developed using Adobe Acrobat software (version Pro DC, Adobe Inc, San José, CA, USA). This data collection tool includes a set of 16 gross-motor and sports-specific tests for balance (*n* = 3; one-leg stance, side-step, and tandem walk), coordination (*n* = 5; hexagon agility test, rapid heel–toe placement, running in place, side-stepping, and split jumps), running, accelerations and decelerations (*n* = 3; 10 m speed skip, 40-m sprint, and stop and go test), jumping (*n* = 4; countermovement jump, four bounds for distance, standing broad jump, and triple hop for distance performed with dominant and non-dominant legs), and change of direction ability (modified agility test). The 16 selected tests are fully described in a study by Roldan et al. [28] and a synthesis of them is reported in Table 2. The test battery was applied at an international CP football competition as a research-specific assessment process and was not part of the standard classification process.

The data collection instrument is structured into three sections (see Supplementary Material File S1). The first section is an index of the document structure following general instructions about how to proceed for its completion. Second, there is a description of the observational categories for each test, including (1) coordination, defined as the ability to voluntarily execute fluid, accurate movements rapidly; (2) balance, defined as the ability to maintain the line of gravity of the body within the base of support with minimal postural sway; (3) active range of movement (AROM), defined as the full movement or optimal potential of a joint or body limb/s, usually its range of flexion and extension, when performing a motor task in dynamic conditions; (4) symmetry, defined as the correspondence and/or movement similarity on opposite sides of a dividing line or plane; and (5) arms impairment, defined as the contribution of the arms to perform the whole movement. At the end of this section, the observer can access the footage with the para-athletes’ performance. Third, the document included 16 independent sections (one per test) with a title, description, reference, and an illustration about the test. The following question was asked for each test: “Which of the following aspects of the athlete’s impairment impact their performance? following the five observation categories with an ordinal scale from 0 to 2 (i.e., “no impact,” “minor impact,” and “major impact,” respectively)”. Some tests were performed for both the left and right sides of the body (i.e., side-step test, rapid heel–toe placement, one-leg stance, and triple hop for distance). In these tests, the observer assessed each body side independently. Afterwards, the observer determined which CP profile/sport class best fit according to the activity limitation exhibited by the para-athlete during the video clips: (1) C5 or moderate spastic diplegia; (2) C6 or moderate ataxia/athetosis; (3) C7 or moderate spastic hemiplegia; (4) C8 or mild impairment of spastic diplegia/hemiplegia, ataxia, or athetosis; or (5) not eligible or no impairment observed during the test performance. The same decision was made at the end of the data collection tool considering the previous inputs for each of the 16 tests.

Before data collection, another three international senior classifiers from Africa, Europe, and Australasia regions reviewed the instrument, and we performed minor edits according to their feedback.

### 2.3. Procedure

To make it easier for classifiers, one video containing the whole battery of tests for each athlete was created (i.e., 16 tests) and was randomly coded (from A01 to A28) to hide the CP profiles/sport classes, which were unknown to the classifiers, i.e., by recording only the important parts of the body (e.g., lower limbs) or blurring the para-athletes’ face in the videos. The video presented the tests in the same order as in the data collection tool, both in regular speed and slow motion. Every video was linked to a single observation form; therefore, every observer completed a total of 28 observations. The classifiers filled in the report as they watched the videos, as explained in the study guidelines. The observation process was individual and self-paced, and the classifiers had a period of three months to complete this task. Contact between observers was not allowed to ensure that responses were personal and not consensual. Classifiers could contact the research team at any time to answer potential questions.

The decisions made by each of the 16 observers for each of the 28 para-athletes were considered for data reduction, leaving out players for whom there is no clear evidence of belonging to one class or the other. For this purpose, the percentage of agreements for the final sport class for each player across the observers was calculated, and a cut-off value of 75% was set for the inclusion of those players’ observations in the data analysis [35,36]. Thus, 21 para-athletes (336 individual observations) were included for data analysis.

### 2.4. Data Analysis

Firstly, we use the forward best-first search to select a subset of variables by considering each variable’s predictive ability and the degree of redundancy between them [37]. Subsets of variables that were highly correlated with each sport class while having low intercorrelation were selected.

A supervised classification technique, C4.5 decision tree (called J48 in WEKA software), was used since it is based on a set of classes known a priori (i.e., in this study, the sport classes) [38]. This technique is an algorithm used to generate a decision tree, which begins with a large group of cases belonging to the known classes. The cases are analysed for patterns that allow the sport classes to be reliably discriminated. These patterns are then expressed as models in the form of decision trees, which can be used to classify new cases [39]. The developed tree was a pruned tree using the default pruning value. The pruning technique was applied to reduce the size of the trees and, at the same time, to avoid complexity [40]. The resultant classification trees were validated via stratified 10-fold cross-validation [41].

We used Cohen’s kappa coefficient [42] and Matthew’s correlations coefficient (MCC) [43] to evaluate the performance of classification models. The Kappa results were interpreted as the following [42]: ≤0, no agreement; 0.01–0.2, none/slight; 0.21–0.40, fair; 0.41–0.60, moderate; 0.61–0.80, substantial; and 0.81–1.00, almost perfect agreement. The MCC results were interpreted as the following [44]: 1 indicates a total positive correlation, 0 is expected for a prediction no better than random, and −1 indicates total disagreement between prediction and observation. In addition, we classified accuracy with the percentage of correct predictions and the area under the receiver operating characteristic curve (AUC-ROC) from 0.5 = non-discrimination to 1 = perfect discrimination. A value below 0.70 is often considered suboptimal, from 0.70 to 0.80 good, and a value of 0.80 or above is excellent [45]. Finally, because an effective model must have high precision (the proportion of instances that are true of a profile divided by the total instances classified as that profile) and high sensitivity (or recall, the proportion of instances classified as a given profile divided by the actual total in that profile), we used F-measures, which is a measure of “effectiveness” and is calculated as a weighted harmonic mean of precision and recall (2 Precision × Recall/(Precision + Recall)). Precision, recall, and F-measure show values between 0.0 (worst possible value) and 1.0 (best or perfect value).

We used the Waikato Environment for Knowledge Analysis (WEKA) open data mining software, version 3.8.3 (The University of Waikato, Hamilton, New Zealand), to perform the statistical analyses.

## 3. Results

### 3.1. Para-Footballer Demographics

Table 3 shows the demographics data of the final sample (*n* = 21) included in the statistical analysis. Considering the percentage of agreements among observer outcomes, seven of the para-athletes were excluded because the agreement between observers was lower than 75%, 13 were categorised as “moderate” impairment (i.e., C5, C6, or C7), and 8 were categorised as “mild” impairment (i.e., C8). The total observation outputs included were 336 (21 × 16) for the overall sample, 64 (4 × 16) for spastic diplegia, 96 (6 × 16) for the ataxic/athetoid profile, and 176 (11 × 16) for spastic hemiplegia. 

### 3.2. Decision Trees Based on Observation Outcomes

**Figure 1:**
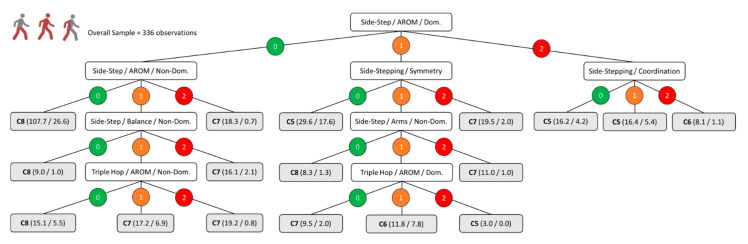
C4.5 decision tree model for the overall sample. The numbers 0, 1, and 2 indicate the impact of the impairment on the specified aspect of the test included in the node as “no impact”, “minor impact”, and “major impact”, respectively. The parentheses indicate the total number of classified observations and the number of incorrect classifications.

**Figure 2:**
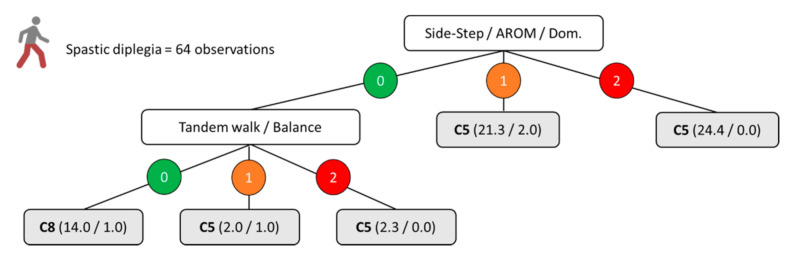
C4.5 decision tree model for the moderate vs. mild profiles of spastic diplegia. The numbers 0, 1, and 2 indicate the impact of the impairment on the specified aspect of the test included in the node as “no impact”, “minor impact”, and “major impact”, respectively. The parentheses indicate the total number of classified observations and the number of incorrect classifications.

**Figure 3:**
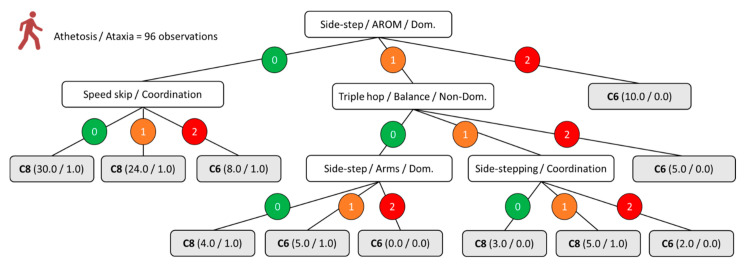
C4.5 decision tree model for the moderate vs. mild profiles of ataxia and athetosis. The numbers 0, 1, and 2 indicate the impact of the impairment on the specified aspect of the test included in the node as “no impact”, “minor impact”, and “major impact”, respectively. The parentheses indicate the total number of classified observations and the number of incorrect classifications.

**Figure 4:**
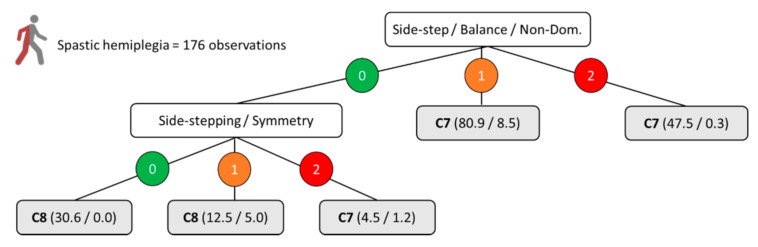
C4.5 decision tree model for the moderate vs. mild profiles of spastic hemiplegia. The numbers 0, 1, and 2 indicate the impact of the impairment on the specified aspect of the test included in the node as “no impact”, “minor impact”, and “major impact”, respectively. The parentheses indicate the total number of classified observations and the number of incorrect classifications.

Figure 1, Figure 2, Figure 3 and Figure 4 show the decision trees developed through the C4.5 algorithm with the overall sample (Figure 1), para-footballers with spastic diplegia (Figure 2), para-footballers with ataxia/athetosis (Figure 3), and para-footballers with spastic hemiplegia (Figure 4). From the battery of 16 tests, the following five tests were included in the decision trees: Side-step, triple hop, side-stepping, tandem walk, and speed skip. In all figures, the decision nodes are represented with white boxes. Each node includes a test, a variable, and one body side under evaluation. The first decision node is the root node (first level), where the best predictor variable is placed. Each decision node has three branches, one for each possible score, i.e., 0 = “no impact” (green colour), 1 = “minor impact” (orange colour), and 2 = “major impact” (red colour). The leaves, emerging from the branches, are presented as grey boxes. Each leaf shows the final class assigned (i.e., C5, C6, C7, or C8), parentheses with the total number of observations registered, and the number representing how many of these were incorrect.

Figure 1 shows the decision tree for the overall sample, with a total of eight decision nodes and 24 branches with 17 leaves. Only three tests were included by the algorithm: Side-step (i.e., AROM and balance performance), triple hop (i.e., AROM performance), and side-stepping (i.e., symmetry and coordination performance), and the AROM of the dominant side, or the less-affected body side, in the side-step test was the best predictor variable for this model.

Figure 2 shows the model for those with moderate (C5) and mild (C8) forms of spastic diplegia, resulting in two nodes and six branches with five leaves. Only two tests were included by the algorithm: Side-step (i.e., AROM performance) and tandem walk (i.e., balance performance). As the model for the overall sample, the AROM of the dominant or less-affected leg during the side-step test was placed in the root node as the best predictor variable.

Concerning the moderate (C6) and mild (C8) profiles of ataxia and athetosis, Figure 3 shows a model of five nodes, 15 branches, and 11 leaves. Only four tests were included by the algorithm: Side-step (i.e., AROM and arms performance), speed skip (i.e., coordination balance), triple hope (i.e., balance performance), and side-stepping (i.e., coordination performance). As with previous profiles, the AROM of the dominant or less-affected body side in the side-step test was the root node of the decision tree model.

For the moderate (C7) and mild (C8) forms of spastic hemiplegia, Figure 4 shows a decision tree model with two nodes, six branches, and five leaves. In this last profile, only two tests were included by the algorithm: Side-step (i.e., balance performance) and side-stepping (i.e., symmetry performance). In this case, the root node was occupied by the observed balance of the non-dominant or more-affected leg in the side-step test.

### 3.3. Effectiveness of the Decision-Tree Models

Table 4 represents the main model’s output characteristics. As can be observed in this table, the decision trees were considered highly accurate with 67.0‒90.9% correctly classified instances. The Kappa coefficient, which analyses the level of agreement with the classifiers, was moderate for the decision tree for HAA and substantial for specific decision trees for each impairment. Table 5 includes the detailed accuracy by CP profile on each decision tree. Observing the outcomes of the MCC and AUC-ROC, the models have a noticeable discrimination prediction, with values from 0.52 to 0.77 and 0.83 to 0.98, respectively.

### 3.4. Parallel Analysis with the Para-Athletes Not Included in the Main Analysis

With the seven para-athletes excluded from the main analysis, the performance of the decision trees was tested (102 total observations) using the sport class assigned for each classifier as the “true” class because we did not have an agreement for these players. While the decision trees for bilateral spasticity or diplegia and unilateral spasticity or hemiplegia achieved a very good and good portion of correctly classified instances (100 and 85.7%, respectively), the decision trees including all the classes and the specific decision tree for players with ataxia or athetosis did not obtain a good portion of correctly classified instances (39.6 and 40%, respectively).

## 4. Discussion

This study aimed to assess the sensitivity of several activity limitation tests and their capacity to discriminate among different levels of impairment in ambulant para-footballers with spastic diplegia, ataxia/athetosis, and spastic hemiplegia. In addition, we searched for a complementary tool for the observation scale designed by Roldan and collaborators [28], intending to facilitate the decision making of sport class allocation in CP football players.

The obtained results have highlighted that the side-step test seems to be the best predictor of the decision tree model, and it is a valid and reliable test for assessing dynamic standing balance in the frontal plane with clinical purposes [29]. Considering that this test is aimed to cover the maximum distance with lateral displacement to the left and the right sides, it seems reasonable to think that the AROM could be the main observation category in this test as it determines motor proficiency. Three observational aspects that can also be evaluated through this test (i.e., AROM of the non-dominant side, balance, and arms impairment) can provide crucial information when deciding on mild (i.e., C8) and moderate (i.e., C7) forms of spastic diplegia. This aspect reinforces the original evidence regarding the application of the side-step test in clinical settings for assessing balance in individuals with spastic hemiplegia [29], which is the most common impairment in people with CP [46] and the most representative profile in CP footballers [14].

Focusing on the overall decision tree model, the side-stepping test was included in two of the nodes, where impaired coordination appears as a key feature for discriminating between moderate profiles of spastic diplegia vs. ataxia/athetosis. In Paralympic classification, reciprocal movements that require inter- (i.e., bilateral) or intra-limb (i.e., unilateral) coordination have been considered as valid methods for assessing impaired coordination in para-athletes with HAA [21,22]. Along these lines, recent research with CP footballers demonstrated that those players with better performance results in the side-stepping test were associated with a greater ability to perform moderate and high-intensity accelerations in a real match, whereas those with lower coordination outcomes performed a lower number of accelerations during the match [47]. On the other hand, symmetry seems to be a relevant feature in discriminating between moderate levels of spastic diplegia and hemiplegia when performing this coordination test. This outcome justifies the reduction in the number of necessary tests to discriminate between unilateral vs. bilateral forms of spasticity [14,28]. However, when symmetry is not enough for discriminating between moderate forms of bilateral and unilateral spasticity, the model suggests using the AROM values of the dominant side in the triple-hop test. This result is in line with Reina et al. [16], who demonstrated that distance covered with unilateral hopping is lower for those with bilateral spasticity when performing with the dominant or less-affected leg (*p* < 0.01; *d* = −1.4, large), but no differences were found for the non-dominant or more-affected legs (*d* = −0.20, trivial). The last node to discriminate between moderate and mild forms of spastic hemiplegia also identifies the AROM of the non-dominant and more affected leg as a key element. Hence, underlying neuromuscular factors such as the increased muscle weakness and affected selective motor control by spasticity would explain the observed AROM differences [48]. However, the effectiveness and the degree of agreement with the observers of this global model was moderate (67.0% correctly classified, Kappa = 0.51), which leads us to recommend the use of specific models for each profile since they are more effective in classification and simpler to apply.

Regarding the players with a CP profile of bilateral spasticity, we found a successful decision-tree model to classify mild (i.e., C8) or moderate (i.e., C5) degrees of impairment (i.e., 89.1% correctly classified instances), with substantial agreement with observers (Kappa = 0.71) and moderate to high accuracy (MCC = 0.715, AUC-ROC = 0.891) and effectiveness (F-Measure = 0.892). Again, the AROM of the dominant side during the side-step test appeared as the best predictor. These results are in line with Roldan et al. [28], who concluded that limited AROM is the most relevant feature in para-athletes with spastic diplegia. Spastic CP is characterised by the development of structural muscle adaptations in response to neural impairments, limiting muscle excursion and leading to the development of contractures and diminished AROM in the affected joints [49,50]. In particular, bilateral spasticity impacts the restriction of limited AROM in lower limbs and the presence of asymmetries as compensatory strategies during running [51,52]. It has also been suggested that gait efficiency is directly influenced by the AROM limitation of the ankle and knee flexion/extension movements, demonstrating the relevance of this parameter on motor function in people with CP [53]. Moreover, these aspects can be found in para-footballers with a high level of impairment and limited walking capacity, where restricted movements of the passive knee extension affect the running speed performance [23]. Furthermore, Connick et al. [54] reported a significant association between a passive range of movement impairments (i.e., maximum thigh flexion and heel pull distance, maximum thigh extension, dorsiflexion lunge, and backward-stepping lunge) with sprint performance (i.e., 60 m maximal sprint) on runners with HAA, indicating the impact of this parameter on sports activity. However, that study combined all the runners with brain impairments in a unique group (T35 = moderate involvement of both legs because of spasticity; T36 = athetosis, ataxia or dystonia affecting all four limbs and the trunk; T37 = spasticity grade 3 or 2 or moderate dystonia, athetosis or ataxia in one half of the body; and T38 = mild impairments of HAA) [6], impeding the comparisons between functional profiles or levels of impairment. On the other hand, para-footballers with bilateral spasticity and higher activity limitation in lower limbs demonstrated a reduced capacity in change of direction ability, acceleration, repeated sprint ability, and maximal speed running during physical assessments in comparison with other functional profiles [18,55]. According to Roldan et al. [28], these AROM limitations can be observed when performing tests such as the MAT (e.g., “Limited hips ROM provokes that the athlete needs to turn the whole body” and “scissor running pattern due to distal spasticity”) or the Stop and Go test (e.g., “The athlete presents ROM limitations like limited ankle dorsiflexion, which makes athletes run using the toes, causing a very small base of support which impacts on the dynamic balance. In consequence, short strides are usually observed”), which required changes of direction and sprinting ability, respectively. In addition, this decision tree model included the impaired balance in the tandem walk test in a second node. Poor balance is a common symptom (i.e., activity limitation) involved in all CP profiles [32] and is deeply studied because of its association with postural control [56]. A zero score on the balance observed in the tandem walk correctly classifies 20.3% of the observations as C8 (i.e., a mild form of the impairment), demonstrating that better balance levels have been related to higher motor function in people with CP [57]. This outcome reinforces the importance of supporting quantitative evidence with qualitative judgements (i.e., observations by the classifiers) to allocate sports classes in para-footballers with CP due to the variability among this population of para-athletes [18].

Four of the 16 observed tests were included in the decision tree model for discriminating between para-footballers with moderate (i.e., C6) and mild (i.e., C8) forms of ataxia or athetosis. The success of this decision tree was good (i.e., 89.1% correctly classified instances), with substantial agreement with observers (Kappa = 0.69) and moderate to high accuracy (MCC = 0.693, and AUC-ROC = 0.842) and effectiveness (F-Measure = 0.864). Following previous studies by Reina et al. [16,17,18] and considering that ataxia and athetosis represent less than 15% of people with CP [58], these impairments have been studied as a unique group. Regarding these two impairments, on the left side of the decision tree, it was found that coordination scores of 0‒1 in the 10 m speed skip test classify the mild forms of ataxia/athetosis in 52/96 observations (i.e., 54.2%), while the remaining 44 observations (i.e., 45.8%) requires other three tests (i.e., triple hop, side-step, and side-stepping). The 10 m speed skip test requires a combination of lower limb power (like the triple hop) and coordination (like the side-stepping) to cover 10 m with the skipping technique as quickly as possible [31], reinforcing that coordination is the most common impaired dimension in this CP profile [28]. Impaired coordination in the side-stepping test would also help at the third level of the model, and this result is in line with the findings by Reina et al. [2], who demonstrated that quantitative scores in this test discriminate between moderate and mild forms of impairment. In this model, it is also remarkable that a “major impact” in the AROM of the dominant body side in the side-step test and on the balance of the non-dominant body side in the triple hop test for distance classifies the moderate forms of this CP profile in 15/96 instances (16.0%), with no presence of disagreements.

Concerning para-footballers with unilateral spasticity, the results suggested that balance in the non-dominant side and symmetry limitations are determinant parameters for class allocation in this subgroup (i.e., moderate = C7 vs. mild = C8 forms of impairment). The success of this decision tree was very high (i.e., 90.9% correctly classified instances), with substantial agreement with observers (Kappa = 0.77) and moderate to high accuracy (MCC = 0.766, AUC-ROC = 0.920) and effectiveness (F-Measure = 0.908). This is a very efficient model since scores of 1 or 2 in the root node (i.e., the observed impaired balance of the non-dominant or more affected leg in the side-step test) correctly classify 128.4 instances (93.1%) as moderate spastic hemiplegia. Recent research describes similar difficulties for maintaining balance stability, indicating differences in control postural strategies in para-footballers with CP during a one-leg stance position with the dominant [59] and when comparing both body sides [32]. Our findings are in line with Reina et al. [14], who demonstrated that the mean velocity of the centre of pressures when performing a one-leg stance is the best predictor for discriminating between different levels of spastic hemiplegia. While our side-step test is a dynamic balance test [29], the transitions during the four steps that comprise the test require balance on one single leg for transferring body weight towards the direction of the displacement. On the other hand, a 0‒1 degree of observed asymmetry in the side-stepping test correctly classifies the mild forms of spastic hemiplegia in 38.1/43.1 instances (88.4%), with a low percentage of disagreements (11.5%) only when the score is 1 (i.e., “moderate impact”). Symmetry has also been suggested as a useful observation variable for para-footballers with a unilateral spasticity profile [28]. The increases in asymmetry in demanding physical tasks, such as running, were linked to impairments associated with upper motor neuron syndrome on the lower limb involvement in CP people [51]. Previous authors showed asymmetrical patterns as a pertinent component to consider in classification assessment due to the implications in the response of the physical and game demands performed by para-footballers with unilateral spasticity [14,16,17,28]. Then, two tests in the frontal plane would be enough for discriminating between moderate and mild forms of spastic hemiplegia.

Finally, some attention should be paid to the higher complexity (i.e., number of nodes and leaves) of the overall model and the model for classifying different levels of athetosis and ataxia. The models found reflect the complexity in classifying the group of para-footballers with ataxia or athetosis due to the characteristics of the impairment and the limited availability of evidence-based tests to assess the impact of the coordination, previously suggested as an impairment-specific relationship [2]. Para-footballers with eligible impairments of ataxia/athetosis exhibit a higher coefficient of variance when performing agility, sprint, and acceleration/deceleration skills, with and without ball dribbling, when compared to the other two CP football profiles of spasticity [18]. Moreover, recent research with this population evidence that this subgroup has greater limitations for both static and dynamic balance, and this fact would influence the performance of basic and specific motor skills differently [32]. Therefore, it is plausible to think that this variability affects the efficiency of the model for this CP subgroup, but also the overall model.

According to Allen et al. [60], an optimal classification system should establish a clear cut-off level maximising sensitivity to adequately identify levels of the impact of the impairment on the sports performance. In other words, having a robust classification system means identifying the essential aspects that will determine players’ allocation in their correct sport classes (i.e., true positive), while avoiding players erroneously excluded for that sport class (i.e., false negative). Our findings support the utility of the proposed observation tool to identify the impact of the impairment on different functional capacities, favouring guidance in decision-making during the classification process of ambulant para-footballers with CP. In this sense, the specific models for each impairment maximise the discriminative capacity, showing high values of precision (0.864–0.908), sensitivity (recall: 0.865–0.909), and effectiveness (F-measure: 0.864–0.908). However, to the best of our knowledge, this is the first study to report decision-tree analysis for supporting decision-making when classifying para-footballers with HAA impairments. These findings are in line with the recommendations for evidence-based classification practices, supporting class allocation based on the extent of activity limitation resulting from the impairment and generated in statistical methods with a scientific approach [3]. CP football introduced a new classification system that involves specific HAA measurements along with sport-specific tests separating the players into the following functional profiles: Profile A (bilateral spasticity), profile B (dyskinesia or ataxia), and profile C (unilateral spasticity) [8]. This study reinforces the key observational features for these profiles and their usefulness for classification purposes, providing guidelines to the classifiers for distinguishing between impairment profiles of CP footballers.

Some study limitations should be mentioned. First, the number of observations included in the data analysis, especially because the number of players was reduced from 28 to 21. At least 20 instances nested in each higher level (para-athletes in our case) are recommended [61,62], and this study only has a maximum of 16 instances per player due to the available international classifiers with the required inclusion criteria. Second, the observation tool was only designed based on the participation of international male CP para-athletes with a high level of performance. Hence, future studies should consider expanding the spectrum of participants to players at the national level and female gender. Third, while representative of this team para-sport, there is no homogeneity in the proportion of the observed CP profiles, there being an over-representation of those with spastic hemiplegia. Fourth, while it is common to study ataxia and athetosis CP profiles as a single group [20,21,22,23], future research should distinguish between them as per the suggested impairment-specific associations between eligible impairment and activity limitation or para-sports performance. In this vein, clinical and objective measurements for assessing the eligible impairments for this para-sport (i.e., HAA) would also be considered in future research.

## 5. Conclusions

In summary, the observation tool based on the impact of specific impairment measurements of HAA permits the determination of which assessment is more appropriate for discriminating between functional profiles in para-footballers with CP. The proposed models are more effective when they are conducted with specific CP profiles, especially those for bilateral and unilateral spasticity, and more research needs to be done on appropriate tests/analyses for those with ataxia and athetosis. In the context of the evidence-based classification system for CP football, decision tree analysis provides reference descriptors and values to support classifiers in decision-making and categorisation of different sport classes. In addition, this study provides new evidence about which activity tests are more efficient for classifying different levels of impairment, optimising the assessment methods and the time required for classification. Further studies should consider other types of statistical methods or prediction tools, such as regression analysis, to adequately explore class assignation and to determine the impairment effect on para-footballer sport performance. In addition, it could also be interesting to develop an instrument to analyse observations during the competition, in line with the presented proposal, to help classifiers determine reliable cut-off points and to support class allocation in this part of the process [7].

## Figures and Tables

**Table 1:** Characteristics of the international classifiers (i.e., observers).

	Physician	Physiotherapist	Sports Technician	Overall
Sex (M/F)	2/0	3/5	3/3	8/8
Age (yr)	53.0 ± 11.3	47.8 ± 11.1	45.4 ± 14.4	47.7 ± 11.7
Occupational Career (yr)	18.0 ± 2.8	26.3 ± 11.8	19.8 ± 11.8	22.5 ± 10.9
National Classifier (yr)	18.0 ± 2.8	14.8 ± 10.5	14.8 ± 11.1	15.3 ± 9.4
International Career (yr)	10.5 ± 6.4	8.0 ± 3.8	5.8 ± 3.1	7.5 ± 4.0

M = male, F = female, yr = years, mean ± SD.

**Table 2:** Activity limitation tests performed by the para-footballers.

Test		Activity Limitation	Equipment	Protocol	Outcome
1	Side-Step	Dynamic balance	Tape measure	The side-step test is performed barefoot without any support and is measured on both sides. A starting line and a 10 m line perpendicular to this are marked on the floor. The athlete performed the test in a standing position with the legs and feet together on the starting line; in principle, the feet make contact when in this position. They then performed five repetitions of side-steps, attempting to step as wide as possible. They did not support their bodies with their arms, nor did they jump [29].	The total distance covered in meters is standardized by using the leg length (distance between the anterior superior iliac spine and the medial malleolus).
2	Rapid Heel-Toe Placement	Coordination	Stopwatch (Casio HS-80TW-1EF). Contact mat (Tapeswitch CVP-2335)	The athlete sits barefoot on a chair and tries to touch the corners of a 20 × 30 cm rectangle on the floor. The athlete alternates heel and toe in each corner, first left-to-right (clockwise) then around right-to-left (anticlockwise). The test is performed twice, with the left foot and with the right foot from the bottom right and left corners, respectively [30].	Time (s) to complete the fastest two trials and the number of incorrect on corners is also recorded.
3	Split Jumps (SJ)	Coordination	Contact mat (Tapeswitch CVP-2335). Marker tape	The player stood with legs slightly apart and one in front of the other. The athlete then jumped over a line by changing the leg position (Left in front, jump changing to Right in front). The arms were simultaneously moved contra-lateral to the legs [31].	Time (s) to complete 25 cycles, and the number of line touches. The best trial is used for analyses.
4	Side-Stepping (SS)	Coordination	Contact mat (Tapeswitch CVP-2335). Marker tape	The player is requested to stand with the legs slightly apart between two lines separated at 40 cm, placing the border of the contact mat on one side. At the tester command, the player jumps over the lines performing symmetrically legs abduction-abduction (open-and-close movements) during 15 repetitions (i.e., cycles). The arms can be moved in a freeway [31].	Time (s) to complete 15 cycles. The best trial is used for analyses.
5	Running in Place	Coordination	Contact mat (Tapeswitch CVP-2335)	Participant stood with both feet next to each other. Participant ran on the same spot as fast as possible for 25 cycles. A cycle is right foot contact to next right foot contact. Tester said stop when 25 correct cycles were completed. Tester counted down: “Ready, Set, GO”. The tester counted the correct cycles out loud, if there is an incorrect one, the tester repeated the same number until the next correct cycle and counting upwards resumed [31].	Time (s) needed to complete 25 correct cycles.
6	Tandem Walk (TW)	Dynamic balance	Stopwatch (Casio HS-80TW-1EF). Marker tape	The player walks barefoot heel to toe along a 5 m line as fast as possible and with the best accuracy, with both arms crossed in front of the chest [32].	Time (s) to complete 10 correct steps and the time to complete 5 m. The best trial is used for analyses.
7	One-Leg Stance (OLS)	Static balance	Stopwatch (Casio HS-80TW-1EF)	The player is barefooted on a spot. Before raising one leg off the floor, participant folded their arms across the chest. The stopwatch started as soon as the player lifted the foot off the floor. The player focused on a spot on the wall at eye level throughout the test. The test was ended when the footballer did any of the following: 1. Uncrossed or used arms to maintain balance; 2. touched the floor with the raised foot; 3. moved the weight-bearing foot; 4. exceeded maximum duration of 20 s [32].	Time (s) keeping the balance with the dominant (OLSD) and the non-dominant (OLSND) legs. The best trial is used for analyses.
8	Counter-Movement Jump (CMJ)	Jumping capability (Vertical)	Leg stiffness device (Opto Jump NextTM, Microgate)	Participants stood on a marked area (force platform) and, in their own time, jumped as high as they could, landing on both feet. Familiarization included standardized instructions, and participants placed their hands on the hips. Three attempts were conducted, and the best score recorded [16].	Jumping height (cm). The best trial is used for analyses.
9	Standing Broad Jump (SBJ)	Jumping capability (Horizontal)	Tape measure	Participants stood on a line and, in their own time, jumped as far forward as they could, and landed on both feet. Familiarization included standardized instructions, and participants could use the stretch-shorten cycle and their arms to increase jump distance [31].	Standardized score (distance/height) for the dominant (THD) and the non-dominant (THND) legs (in m/m).
10	Modified Agility Test (MAT)	Change of Direction Ability	Time gates (GlobusTM). Cones.	Participants were asked to begin 0.5 m behind the starting line and sprint forward 5 m, as fast as possible, touching the cone (30 cm) with one hand, and in this order, moving laterally (2.5 m) without crossing the feet to touch the top of cone at left; then moving laterally (5 m) to touch the top of cone at right; then moving laterally (2.5 m) to touch the top of cone at left, and finally return backward (5 m) to starting line. The total distance covered is 20 m [17].	Time to complete the course (s). The fastest trial is used for data analyses.
11	Hexagon Agility Test	Coordination	Stopwatch (Casio HS-80TW-1EF). Marker tape	A hexagon with 60 cm sides and 120-degree angles is marked on a hard-surface floor. The test begins with the subject standing on a tape strip placed in the middle of the hexagon (starting location) and performs double-leg hopping from the centre of the hexagon over each side and back to the centre in a clockwise direction until the participant goes around the hexagon 3 times and returns to the centre (18 jumps) [33].	Time (s) to complete 3 revolutions around the hexagon.
12	Triple Hop for Distance(TH)	Jumping capability (Horizontal)	Tape measure	The triple hop involved participants performing three consecutive maximal hops and landing on the same leg. The jumps could be assisted by swinging the upper body and arms. Distance (m) is measured from the start line to the rear of the foot upon final landing. Besides, the participant’s height is required for the standardised score [16].	Standardized score (distance/height) for the dominant (THD) and the non-dominant (THND) legs (in m/m).
13	Four Bounds for Distance (4B)	Jumping capability (Horizontal)	Tape measure	Participants started on a marked line and were instructed to cover the maximum possible distance in four consecutive single-leg bounds from a standing start. The first bound was from their non-preferred leg, landing on their outstretched preferred leg. Using forward momentum to continue the movement, the second bound was conducted as they leapt from their preferred leg to their non-preferred leg. This pattern was repeated for a total of 4 bounds. Distance is measured from the starting line to the heel strike of the fourth bound (m) [16,31].	Standardized score (distance/height) for the dominant (THD) and the non-dominant (THND) legs (in m/m).
14	10 m Speed Skip	Running + Coordination	Time gates (GlobusTM)	Markers were placed at 0, 10 and 20 m with pairs of infrared timing light gates positioned at the 10 and 20 m markers. Participants performed the skip—a hop-step—hop pattern were allowed to practice until they could complete the pattern over 10 m. Participants accelerated over the first 10 m so that they were at top speed when they reach the first light gate (10 m) and maintained top-speed as they moved through to the second gate (20 m) [31].	Time (s) to move from 10 to 20 m was recorded.
15	Stop & Go Test	Accelerations and Decelerations	Time gates (GlobusTM)Contact mat (Tapeswitch CVP-2335)	The athlete stood without support behind the starting line and started to run at the researcher’s signal. The athlete ran to a mat (10 m) and stopped completely on the mat with both feet. After the first contact, the athlete remained on the mat for 2 s until a beep sounded. Immediately at the sound they ran again to the next mat (10 m) and stopped again until the next beep, and then continued to the final mark at 10 m from the second mat. Total distance = 30 m [34].	Time (s), measured with time gates to the first mat (at 10 m), second mat (at 20 m), last gate (at 30 m), total time (30 m distance)
16	40 m Sprint	Acceleration + Sprint	Time gates (GlobusTM)	The player ran at maximum speed from a standing start to 40 m. Timing light gates are positioned at 0, 10, 25 and 40 m [31].	Time (s) to complete 10, 25 and 40 m. The best trial is used for analyses.

**Table 3:** Characteristics of the para-athletes with cerebral palsy included for data analysis.

	Bilateral Spasticity or Diplegia	Ataxia or Athetosis	Unilateral Spasticity or Hemiplegia	Overall
*n* (Moderate/Mild)	4 (3/1)	6 (3/3)	11 (7/4)	21 (13/8)
Age (yr)	28.6 ± 8.1	24.2 ± 7.2	25.2 ± 5.2	25.5 ± 6.2
Height (cm)	173.8 ± 9.1	178.5 ± 7.5	174.1 ± 10.3	175.3 ± 9.2
Body weight (kg)	70.2 ± 9.6	76.1 ± 8.4	67.0 ± 9.0	70.1 ± 9.3
BMI (kg·m^−2^)	23.2 ± 2.5	23.7 ± 2.5	22.1 ± 1.2	22.7 ± 2.0
Experience (yr)	6.5 ± 5.8	9.8 ± 5.2	14.3 ± 7.4	11.5 ± 7.0

yr = years, cm = centimetres, kg = kilograms, BMI = body mass index, mean ± SD.

**Table 4:** Main models characteristics.

	Overall	Spastic Diplegia	Ataxia or Athetosis	Spastic Hemiplegia
Correctly classified instances	67.0%	89.1%	86.5%	90.9%
Incorrectly classified instances	33.0%	10.9%	13.5%	9.1%
Kappa coefficient	0.51	0.71	0.69	0.77

**Table 5:** Weighted average accuracy for every C4.5 decision-tree.

	TP Rate	FP Rate	Precision	Recall	F Measure	MCC	AUC-ROC	PRC Area
Overall	0.670	0.155	0.657	0.670	0.662	0.516	0.825	0.667
Spastic diplegia	0.891	0.161	0.893	0.891	0.892	0.715	0.874	0.891
Ataxia or athetosis	0.865	0.177	0.864	0.865	0.864	0.693	0.875	0.842
Spastic hemiplegia	0.909	0.164	0.908	0.909	0.908	0.766	0.980	0.920

TP Rate: Tate of true positives (instances correctly classified as a given class). FP Rate: Tate of false positives (instances falsely classified as a given profile). Precision: The proportion of instances that are true of a profile divided by the total instances classified as that profile. Recall: The proportion of instances classified as a given profile divided by the actual total in that profile (equivalent to TP rate). F-Measure: A combined measure for precision and recall calculated as 2 × Precision × Recall/(Precision + Recall). MCC: Matthews’ correlation coefficient. AUC-ROC: Area under the receiver operating characteristic curve. PRC: Precision-recall curves.

## Data Availability

Data collection form is included as supplementary file of this study.

## Supplementary Materials

The following is available online at https://www.mdpi.com/article/10.3390/ijerph18084320/s1, File S1: data collection form used by observers to categorize the impact of the impairment exhibited by people with different CP profiles of spastic diplegia, athetosis/ataxia, or spastic hemiplegia when performing the tests battery requiring jumping, sprinting, change of direction, coordination, or balance skills.
