# Supplementary material for: Using Decision Trees to Support Classifiers’ Decision-Making about Activity Limitation of Cerebral Palsy Footballers
Source: Int J Environ Res Public Health. 2021 Apr 19;18(8):4320. doi: 10.3390/ijerph18084320 (PMC8074122; doi:10.3390/ijerph18084320)
Supplement: Supplementary file 1 [file ijerph-18-04320-s001.zip › ijerph-1145669-supplementary.pdf]

supported by AGITOS Foundation

centro de investigación del deporte — Universidad Miguel Hernández de Elche

UNIVERSITAS Miguel Hernández

Improving reliability and validity of current classification methods for athletes in classes T35 to T38

Instructions and Data Collection Form

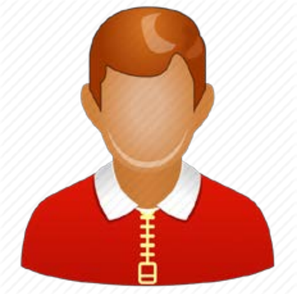

Athlete ID:

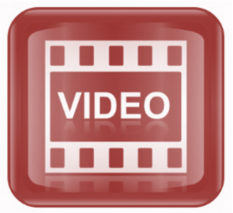

INDEX	PAGE
INSTRUCTIONS	2
HOW TO USE DATA COLL. FORM	4
DATA COLLECTION FORM	
1. Side-Step test	7
2. Rapid Heel-Toe Placement	8
3. Split Jumps	10
4. Side-Stepping	12
5. Running in place	14
6. Tandem Walk	16
7. One Leg Stance	17
8. Countermovement Jump	19
9. Standing Broad Jump	20
10. MAT Test	22
11. Hexagon Hop Test	25
12. Triple Hop for Distance	27
13. Four Bounds for Distance	28
14. 10m Speed Skip	30
15. Stop and go	32
16. 40 m Sprint	34
FINAL OUTCOME	37

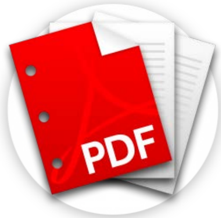

Instructions

Before you start:

1. Re-read the Participant Information Document, to remind yourself about:
   a. Your Role in the Project (i.e. what you need to do)
   b. the project materials
2. Read through all the instructions in this form and make sure you understand the process. Contact me if you have any questions (rreina@goumh.es)

Materials:

A reminder: data has been collected on 28 athletes. All are CP football players who competed at CPISRA ICUP 2013. Each athlete has been allocated an identification number from A01 to A28. Each athlete has a folder on dropbox which has their athlete ID on it (A01-A28) with two files in it – data collection form and a copy of the current class profiles. Videos are linked to the video icon in each data collection form (to can work on-line by video-streaming) or you can access through a Dropbox link (download and work off-line). The link to video files has been send by e-mail, together the link tou your personal Dropbox folder.

Open the folder for an athlete, open the two documents and the video (internet browser or video player), and arrange them so you can see all three at once. One way you could do this is to arrange them on your screen as the example below shows. However, if your screen is very small, this might not work, so an alternative would be to just have the video file and the data collection form open and print the class profiles and the definitions of the observation categories to manage it more easily. Another option would be to use 2 screens simultaneously (e.g., play the video through your TV, have the data collection form open in your PC and have printed copies of the class profiles).

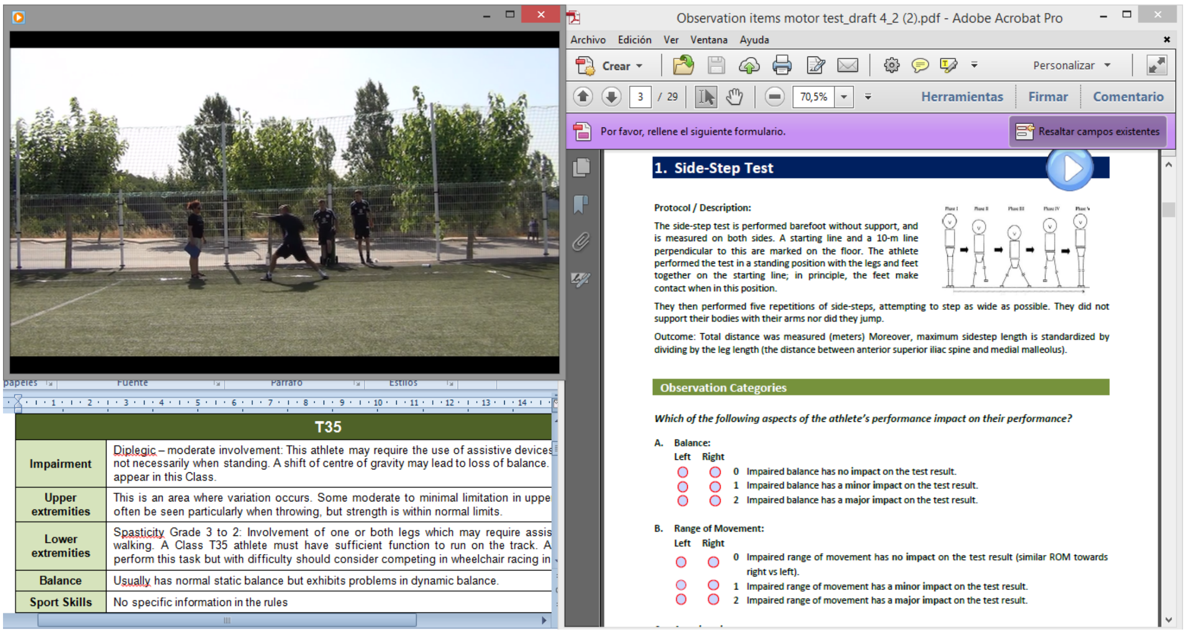

To complete this process:

- Play the first test on the video (sidestep test) and complete the questions on the data collection form for the side-step test.

How to use the Data Collection Form?

Each Data Collection Form includes the following information about each test:

a) *Description of the test protocol.* You can get information about the instructions given to the athletes for each test.

b) Measured outcome. You can get information about the measured units used in each test.

c) *Picture or diagram*, showing each test.

d) *List of observation categories*. These categories evaluate your opinion about the impact (**from 0 to 2**) of the following performance characteristics on the test outcome:

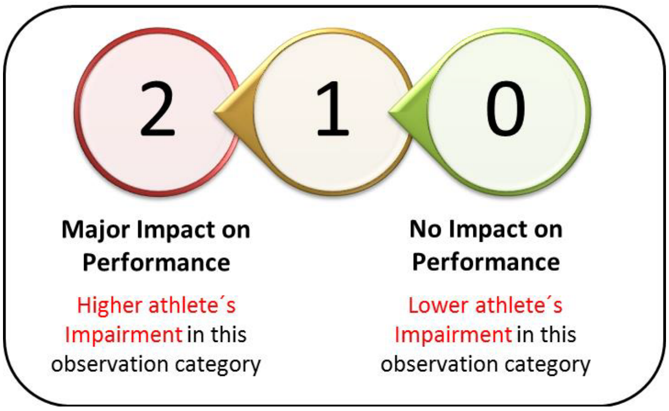

   a. ***Coordination,*** defined as the ability to voluntarily execute fluid, accurate movements rapidly.
   b. ***Balance,*** defined as the

   from centre of mass) of a body within the base of support with minimal postural sway.
   c. ***Symmetry,*** *defined as the* correspondence and/or m vement similarity on opposite sides of a dividing line or plane.
   d. ***Range of movement***, defined as the full movement or optimal potential of a joint, usually its range of flexion and extension.
   e. ***Arm impairment***, defined as the contribution of the arms to perform the whole movement.

Data Collection Form

1. Side-Step Test

Protocol / Description:

The side-step test is performed barefoot without support, and is measured on both sides. A starting line and a 10-m line perpendicular to this are marked on the floor. The athlete performed the test in a standing position with the legs and feet together on the starting line; in principle, the feet make contact when in this position.

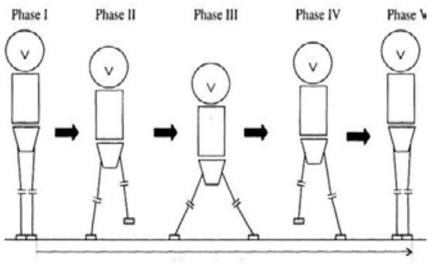

They then performed five repetitions of side-steps, attempting to step as wide as possible. They did not support their bodies with their arms nor did they jump.

Outcome: Total distance was measured (meters)

Observation Categories

Which of the following aspects of the athlete's performance impact on their performance?

A. Balance:

**Left**	**Right**		
◯	◯	**0**	Impaired balance has **no impact** on the test result.
◯	◯	**1**	Impaired balance has a **minor impact** on the test result.
◯	◯	**2**	Impaired balance has a **major impact** on the test result.

B. Range of Movement:

**Left**	**Right**		
◯	◯	**0**	Impaired range of movement has **no impact** on the test result (similar ROM towards right vs left).
◯	◯	**1**	Impaired range of movement has a **minor impact** on the test result.
◯	◯	**2**	Impaired range of movement has a **major impact** on the test result.

C. Arms impairment:

**Left**	**Right**		
◯	◯	**0**	Athlete is able to **effectively use arms** to assist with the test performance.
◯	◯	**1**	Athlete is able to **use arms a little bit** to assist with test performance.
◯	◯	**2**	Athlete **does not use arms** to assist with the test performance.

2. Rapid Heel-Toe Placement

Protocol / Description:

The athlete sat barefoot on a chair and tried to touch the corners of 20 x 30 cm rectangle on floor. Athlete alternated heel and toe in each corner, first left to right (clockwise) then around right to left (anticlockwise). With the left foot, the athlete started from the bottom left corner, and with the right foot from bottom right corner.

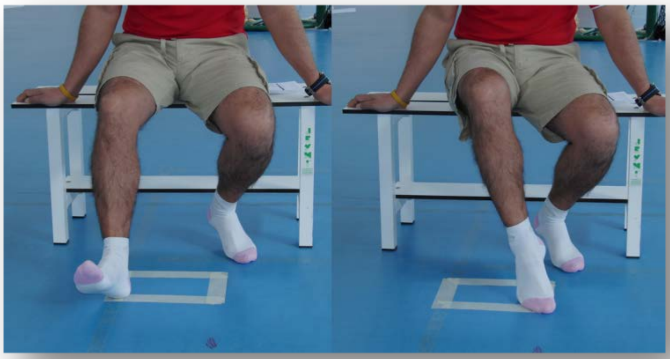

Test Outcome: Time (s) was measured for the fastest two trials, and number of incorrect contacts on corners was recorded.

Source: Proposed by an anonymous expert (2012) for Bicici, S., Tweedy, S. & Vanlandewijck, Y. (2012). Development of a test battery for improving classification reliability for ambulant athletes affected by hypertonia, ataxia or athetosis. KU Leuven: Unpublished Master Thesis.

Observation Categories

Athlete must physically touch each corner with his heel and/or toe. If heel/toe missed corner, this is not a valid touch. Therefore the athlete should make maximum 14 touches on the corners.

Evaluate athlete´s performance to touch (heel and toe contact) the corners…

A. Coordination

Left	Right		
○	○	**0**	Good heel and toe touch.
○	○	**1**	One touch is better than other one (heel < > toe).
○	○	**2**	No precision - touch square corners.

B. Distal Range of Movement:

Left	Right		
○	○	**0**	The athlete had a **good** plantar flexion and dorsi flexion and that these factors did not influence the test result.
○	○	**1**	Lack of ankle plantar flexion and dorsi flexion had a **minor impact** on the test outcome (they had better ankle plantar flexion and dorsi flexion would get a better test result).
○	○	**2**	Lack of ankle plantar flexion and dorsi flexion had a **major impact** on the test outcome.

C. Proximal Range of Movement:

Left	Right		
○	○	**0**	Loss of range of movement in proximal joints (knee/hip) had **no impact** (i.e. range of movement was normal).
○	○	**1**	Loss of range of movement in proximal joints (knee/hip) had a **minor impact** on test outcome.
○	○	**2**	Loss of range of movement in proximal joints (knee/hip) had a **major impact** on test outcome.

3. Split Jumps

Athlete stood with legs slightly apart and one in front of the other. The athlete then jumped over a line by changing the leg position (Left in front, jump changing to Right in front). The arms were simultaneously moved contra-lateral to the legs.

Outcome: Time needed to complete 25 correct cycles, (seconds), and number of line touches.

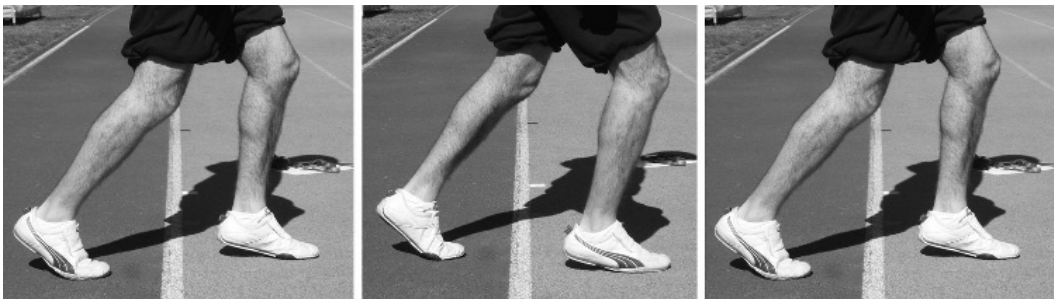

Source: Beckman, E.M. & Tweedy, S.M. (2009) Towards evidence-based classification in Paralympic athletics: evaluating the validity of activity limitation tests for use in classification of Paralympic running events. *British Journal of Sports Medicine*, 43, 1067-1072

Observation Categories

What of the next aspects of the athlete's performance have more impact on their performance?

A. Coordination:

- ○ **0** Impaired coordination had **no impact** on the test outcome (i.e. athletes show good coordination and fluency).
- ○ **1** Impaired coordination had a **minor impact** on the test outcome (i.e. sometimes lose coordination but able to continue after a quick stop or decrease in speed).
- ○ **2** Impaired coordination had a **major impact** on the test outcome (i.e. movement fluency breaks down continuously).

B. Balance:

- ○ **0** Impaired balance had **no impact** on the test outcome (i.e. postural sway is minimal).
- ○ **1** Impaired balance had a **minor impact** on the test outcome.
- ○ **2** Impaired balance had a **major impact** on the test outcome (i.e. athlete has difficulties to keep the starting position).

C. Symmetry:

- ○ **0** Impaired symmetry had **no impact** on the test outcome (i.e. no clear difference right vs left).
- ○ **1** Impaired symmetry had a **minor impact** on the test outcome.
- ○ **2** Impaired symmetry had a **major impact** on the test outcome (i.e. clear difference right vs left).

D. Range of Movement:

- ○ **0** Impaired range of movement had **no impact** on the test outcome.
- ○ **1** Impaired range of movement had a **minor impact** on the test outcome.
- ○ **2** Impaired range of movement had a **major impact** on the test outcome.

E. Arms impairment:

- ○ **0** Athlete is able to **effectively use arms** to assist with the test performance (i.e. arms movement is contralateral regarding legs).
- ○ **1** Arms impairment had a **minor impact** on the test outcome.
- ○ **2** Arms impairment had a **major impact** on the test outcome.

4. Side Stepping

Protocol / Description:

Athlete stood with legs slightly apart between two lines separated at 40 cm then jumped over the line performing symmetrical abduction-abduction of the legs (open-close). The arms could be moved freely.

Outcome: Time needed to complete 15 correct cycles (seconds), and number of invalid trials (both feet did not step outside the line or inside the line)

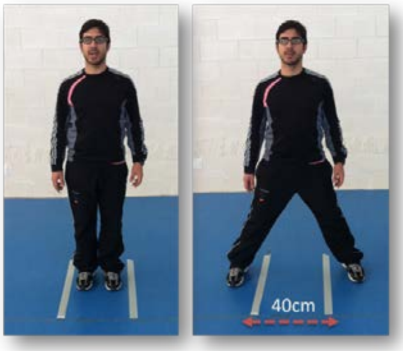

Observation Categories

What of the next aspects of the athletes´ performance have more impact on their performance?

A. Coordination:

- ○ **0** Impaired coordination had **no impact** on the test outcome (i.e. athletes shows good coordination and fluency).
- ○ **1** Impaired coordination had a **minor impact** on the test outcome (i.e. sometimes loses coordination but able to continue after a quick stop or speed decrease).
- ○ **2** Impaired coordination had a **major impact** on the test outcome (i.e. movement fluency breaks down continuously).

B. Balance:

- ○ **0** Impaired balance had **no impact** on the test outcome (i.e. postural sway is minimal).
- ○ **1** Impaired balance had a **minor impact** on the test outcome.
- ○ **3** Impaired balance had a **major impact** on the test outcome (i.e. athlete has difficulties to keep the starting position).

C. Symmetry:

- ○ **0** Impaired symmetry had **no impact** on the test outcome.
- ○ **1** Impaired symmetry had a **minor impact** on the test outcome.
- ○ **2** Impaired symmetry had a **major impact** on the test outcome.

D. Range of Movement:

- ○ **0** Impaired range of movement had **no impact** on the test outcome.
- ○ **1** Impaired range of movement had a **minor impact** on the test outcome.
- ○ **2** Impaired range of movement had a **major impact** on the test outcome.

5. Running in Place

Protocol / Description:

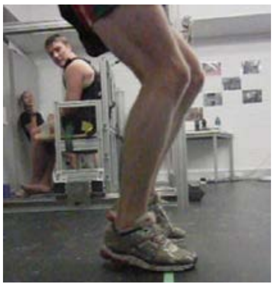

Participant stood with both feet next to each other. Participant ran on the same spot as fast as possible for 25 cycles. A cycle is right foot contact to next right foot contact. Tester said stop when 25 correct cycles were completed. Tester counted down: “Ready, Set, GO”. Tester counted the correct cycles out loud, if there is an incorrect one, tester repeated the same number until the next correct cycle and counting upwards resumed.

Outcome: Time needed to complete 25 correct cycles (seconds)

Source: Beckman, E.M. & Tweedy, S.M. (2009) Towards evidence-based classification in Paralympic athletics: evaluating the validity of activity limitation tests for use in classification of Paralympic running events. *British Journal of Sports Medicine*, 43, 1067-1072

Observation Categories

What of the next aspects of the athletes’ performance have more impact on their performance?

A. Coordination:

- ○ **0** Impaired coordination had **no impact** on the test outcome.
- ○ **1** Impaired coordination had a **minor impact** on the test outcome.
- ○ **2** Impaired coordination had a **major impact** on the test outcome.

B. Balance:

- ○ **0** Impaired balance had **no impact** on the test outcome.
- ○ **1** Impaired balance had a **minor impact** on the test outcome.
- ○ **2** Impaired balance had a **major impact** on the test outcome.

C. Symmetry:

- ○ **0** Impaired symmetry had **no impact** on the test outcome.
- ○ **1** Impaired symmetry had a **minor impact** on the test outcome.
- ○ **2** Impaired symmetry had a **major impact** on the test outcome.

6. Tandem Walk

Protocol / Description:

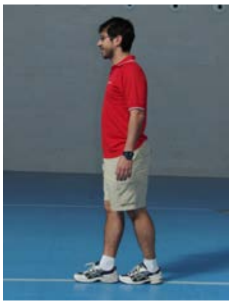

Participant walked barefoot, heel to toe, along a line as fast as possible and with best accuracy, with both arms crossed in front of chest. Two conditions were applied:

a) Time to complete 10 correct steps (heel needs to touch the toe during each step)
b) Time to complete 5m distance (heel-toe contact is not necessary)

Outcome: Time (seconds)

Source: Proposed by an anonymous expert (2012) for Bicici, S., Tweedy, S. & Vanlandewijck, Y. (2012). Development of a test battery for improving classification reliability for ambulant athletes affected by hypertonia, ataxia or athetosis. KU Leuven: Unpublished Master Thesis.

Observation Categories

What of the next aspects of the athletes' performance have more impact on their performance?

A. Balance:

- ◯ **0** Impaired balance had **no impact** on the test outcome (i.e. posture and control throughout the test was good and the athlete was in control at all times or no requires steps out of the line to complete the test).
- ◯ **1** Impaired balance had a **minor impact** on the test outcome.
- ◯ **2** Impaired balance had a **major impact** on the test outcome (i.e. athlete was frequently out of balance or they had to work hard to recover).

B. Symmetry:

- ◯ **0** Impaired symmetry had **no impact** on the test outcome.
- ◯ **1** Impaired symmetry had a **minor impact** on the test outcome.
- ◯ **2** Impaired symmetry had a **major impact** on the test outcome.

C. Arms impairment:

- ◯ **0** Arms still cross in front chess during the test.
- ◯ **1** Sometimes use the arms to keep position / short arm movements.
- ◯ **2** Requires arms to keep balance / wide arm movements.

7. One Leg Stance

Protocol / Description:

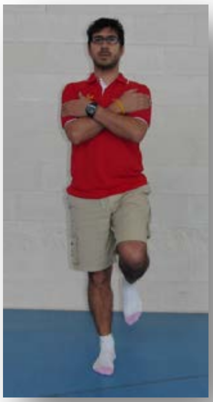

The player stood barefoot on a force platform. Prior to raising one leg off the floor, participants folded their arms across the chest. Stopwatch was started as soon as athlete lifted the foot off the floor. The player focused on a spot on the wall at eye level throughout the test.

Outcome: Mean score of the centre of pressure displacement (cm), measured on a force platform.

Source: Springer, B.A., Marin, R., Cyhan, T., Roberts, H. & Gill, N.W. (2007) Normative values for the Unipedal Stance Test with Eyes Open and Closed, *Journal of Geriatric Physical Therapy*, 30 (1), 8-15.

Observation Categories

What of the next aspects of the athletes' performance have more impact on their performance?

A. Balance:

**Left**	**Right**		
◯	◯	**0**	Impaired balance had **no impact** on the test outcome (i.e. Posture and control throughout the test was good and the athlete was in control at all times).
◯	◯	**1**	Impaired balance had a **minor impact** on the test outcome.
◯	◯	**2**	Impaired balance had a **major impact** on the test outcome (i.e. the athlete was frequently out of balance or they had to work hard to recover.

8. Counter-Movement Jump

Protocol / Description:

Participants stood on a marked area (force platform) and, in their own time, jumped as high as they could, landing on both feet. Familiarization included standardized instructions, and participants placed their hands on the hips.

Three attempts were conducted and best score recorded.

Outcome: Height (cm)

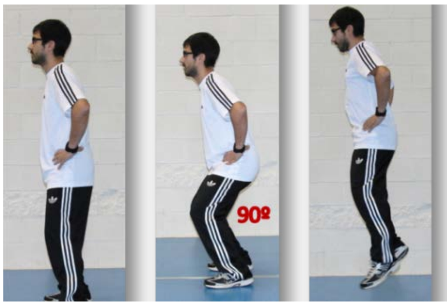

Source: Cámara, J., Grande, I., Mejuto, G., Los Arcos, A., & Yanci, J. (2013). Jump landing characteristics in elite soccer players with Cerebral palsy. *Biology of Sport*, 30(2)

Observation Categories

What of the next aspects of the athletes' performance have more impact on their performance?

A. Coordination:

- ◯ **0** Impaired coordination had **no impact** on the test outcome (i.e. good movement fluency).
- ◯ **1** Impaired coordination had a **minor impact** on the test outcome.
- ◯ **2** Impaired coordination had a **major impact** on the test outcome (i.e. difficulties to move from eccentric to concentric phase).

B. Balance:

- ◯ **0** Impaired balance had **no impact** on the test outcome (i.e. jump is vertical).
- ◯ **1** Impaired balance had a **minor impact** on the test outcome.
- ◯ **2** Impaired balance had a **major impact** on the test outcome (I.e. difficulties to land on the same place than he takes-off).

C. Symmetry:

- ◯ **0** Impaired symmetry had **no impact** on the test outcome (i.e. legs impulse at the same time and power).
- ◯ **1** Impaired symmetry had a **minor impact** on the test outcome (i.e. slight difference right <> left or slight incoordination / power).
- ◯ **2** Impaired symmetry had a **major impact** on the test outcome (i.e. clear differences right <> left or noticeable incoordination /power).

D. Range of Movement:

- ◯ **0** Impaired range of movement had **no impact** on the test outcome.
- ◯ **1** Impaired range of movement had a **minor impact** on the test outcome.
- ◯ **2** Impaired range of movement had a **major impact** on the test outcome.

E. Arms impairment:

- ◯ **0** Hands stay on the hips during the jump.
- ◯ **1** Athlete shows difficulties to keep arms on the hips.
- ◯ **2** Hands come away from the hips.

9. Standing Broad Jump

Protocol / Description:

Participants stood on a line and, in their own time, jumped as far forward as they could, and landed on both feet. Familiarization included standardized instructions, and participants could use the stretch–shorten cycle and their arms to increase jump distance.

Outcome: Distance jumped (cm) is measured from the start line to the heel strike. Distance is divided by height for standardization.

Source: Beckman, E.M. & Tweedy, S.M. (2009) Towards evidence-based classification in Paralympic athletics: evaluating the validity of activity limitation tests for use in classification of Paralympic running events. *British Journal of Sports Medicine*, 43, 1067-1072

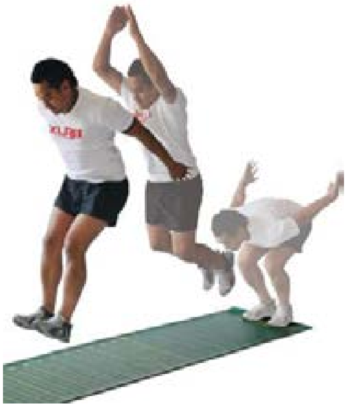

Observation Categories

What of the next aspects of the athletes' performance have more impact on their performance?

A. Coordination:

- ○ **0** Impaired coordination had **no impact** on the test outcome.
- ○ **1** Impaired coordination had a **minor impact** on the test outcome.
- ○ **2** Impaired coordination had a **major impact** on the test outcome.

B. Balance:

- ○ **0** Impaired balance had **no impact** on the test outcome.
- ○ **1** Impaired balance had a minor impact on the test outcome.
- ○ **2** Impaired balance had a major impact on the test outcome.

C. Symmetry:

- ○ **0** Impaired symmetry had **no impact** on the test outcome.
- ○ **1** Impaired symmetry had a **minor impact** on the test outcome.
- ○ **2** Impaired symmetry had a **major impact** on the test outcome.

D. Range of Movement:

- ○ **0** Impaired range of movement had **no impact** on the test outcome.
- ○ **1** Impaired range of movement had a **minor impact** on the test outcome.
- ○ **2** Impaired range of movement had a **major impact** on the test outcome.

E. Arms impairment:

- ○ **0** Athlete is able to **effectively use arms** to assist with the test performance.
- ○ **1** Athlete is able to **use arms a little bit** to assist with test performance.
- ○ **2** Athlete **does not use arms** to assist with the test performance.

10. MAT Test

Protocol / Description:

Participant ran the MAT course as fast as possible with the following directions:

1) A-B movements (5 m): Participants sprinted forward to cone B and touch the top of it with the right hand.
2) B-C movements (2.5 m): Moving laterally without crossing the feet, participants ran to cone C and touched its top with the left hand.
3) C-D movements (5 m): Participants ran laterally to cone D and touched its top with the right hand.
4) D-B movements (2.5 m): Participants moved back to cone B and touched its top with the left hand.
5) B-A movements (5 m): Participants ran backwards to line A.

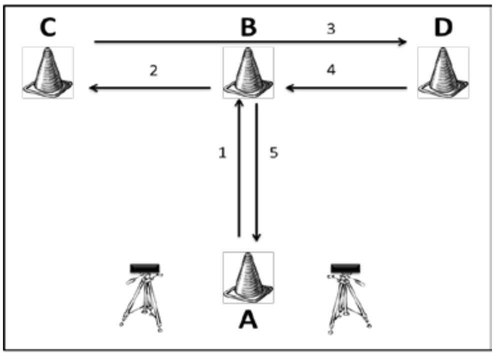

Trials where participants crossed their feet during B-C, C-D and D-B movements, failed to touch the top of the cone, and/or failed to face forward throughout the tasks, were repeated.

Outcome: time (s), measured with time gates.

Source: Yanci, J., Los Arcos, A., Reina, R., Gil, E., y Grande, I. (2014). La agilidad en alumnos de educación primaria: diferencias por edad y sexo. *Revista Internacional de Medicina y Ciencias de la Actividad Física y del Deporte*, *14* (53), 23-35.

Observation Categories

What of the next aspects of the athletes' performance have more impact on their performance?

A. Coordination:

- ◯ **0** Impaired coordination had **no impact** on the test outcome.
- ◯ **1** Impaired coordination had a **minor impact** on the test outcome.
- ◯ **2** Impaired coordination had a **major impact** on the test outcome.

B. Balance:

- ◯ **0** Impaired balance had **no impact** on the test outcome.
- ◯ **1** Impaired balance had a **minor impact** on the test outcome.
- ◯ **2** Impaired balance had a **major impact** on the test outcome.

C. Symmetry:

- ◯ **0** Symmetry had **no impact** on the test outcome.
- ◯ **1** Symmetry had a **minor impact** on the test outcome.
- ◯ **2** Symmetry had a **major impact** on the test outcome.

D. Range of Movement:

- ◯ **0** Range of movement had **no impact** on the test outcome.
- ◯ **1** Range of movement had a **minor impact** on the test outcome.
- ◯ **2** Range of movement had a **major impact** on the test outcome.

E. Arms impairment:

- ◯ **0** Athlete is able to **effectively use arms** to assist with the test performance.
- ◯ **1** Athlete is able to **use arms a little bit** to assist with test performance.
- ◯ **2** Athlete **does not use arms** to assist with the test performance.

11. Hexagon Jump Test

Protocol / Description:

A hexagon with 60cm sides and 120-degree angles is marked with tape on a hard-surface floor with tape strip in the middle to mark the starting position. The test began with the subject standing on the tape strip placed in the middle of the hexagon, which marks the starting location. The tester gave the command ''Ready, go'' and starts the stopwatch. On the ''Go'' command, the participant began jumping (both legs) from the centre of the hexagon over each side and back to the centre in a clockwise direction until the participant went around the hexagon 3 times and returned to the centre (18 jumps). The stopwatch was stopped once the participant returned to the centre mark after 3 revolutions around the hexagon.

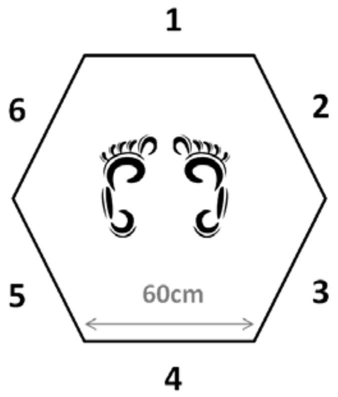

The participants faced the same direction during the course of the test, and the feet couldn't land on the taped edges of the hexagon or the trial was stopped and restarted. The participants were instructed to perform the test as fast as they could. No verbal inducements or encouragement were provided during the testing.

Outcome: Time to complete 3 revolutions (seconds)

Source: Beekhuizen, K.S., Davis, M.D., Kolber, M.J. & Cheng, M.S.S. (2009) Test-retest reliability and minimal detectable change of the hexagon agility Test. *Journal of Strength and Conditioning Research*, 23, 2167-2171.

Observation Categories

What of the next aspects of the athletes' performance have more impact on their performance?

A. Coordination:

- ◯ **0** Impaired coordination had **no impact** on the test outcome.
- ◯ **1** Impaired coordination had a **minor impact** on the test outcome.
- ◯ **2** Impaired coordination had a **major impact** on the test outcome.

B. Balance:

- ◯ **0** Impaired balance had **no impact** on the test outcome.
- ◯ **1** Impaired balance had a **minor impact** on the test outcome.
- ◯ **2** Impaired balance had a **major impact** on the test outcome.

C. Symmetry:

- ◯ **0** Impaired symmetry had **no impact** on the test outcome.
- ◯ **1** Impaired symmetry had a **minor impact** on the test outcome.
- ◯ **2** Impaired symmetry had a **major impact** on the test outcome.

D. Range of Movement:

- ◯ **0** Impaired range of movement had **no impact** on the test outcome.
- ◯ **1** Impaired range of movement had a **minor impact** on the test outcome.
- ◯ **2** Impaired range of movement had a **major impact** on the test outcome.

12. Triple Hop for Distance

Protocol / Description:

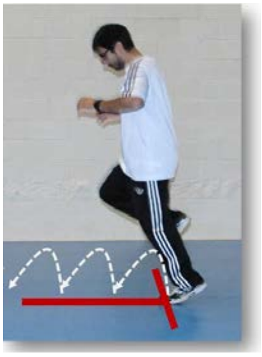

Subjects were instructed to stand on one leg and perform 3 consecutive hops as far as possible, landing on the same leg.

Both limbs were tested (2 trials with each leg), and no restrictions were given to subjects regarding the use of arm movement (two valid trials with each leg)

Outcome: The total distance for 3 consecutive hops is recorded (m)

Munro, AG and Herrington, LC. (2011) Between-session reliability of four hop tests and the agility T test. *Journal of Strength and Conditioning Research*, 25(5), 1470-1477

Observation Categories

What of the next aspects of the athletes' performance have more impact on their performance?

A. Coordination:

Left	Right		
○	○	**0**	Impaired coordination had **no impact** on the test outcome.
○	○	**1**	Impaired coordination had a **minor impact** on the test outcome.
○	○	**2**	Impaired coordination had a **major impact** on the test outcome.

B. Balance:

Left	Right		
○	○	**0**	Impaired balance had **no impact** on the test outcome.
○	○	**1**	Impaired balance had a **minor impact** on the test outcome.
○	○	**2**	Impaired balance had a **major impact** on the test outcome.

C. Range of Movement:

Left	Right		
○	○	**0**	Impaired range of movement had **no impact** on the test outcome.
○	○	**1**	Impaired range of movement had a **minor impact** on the test outcome.
○	○	**2**	Impaired range of movement had a **major impact** on the test outcome.

D. Arms impairment:

Left	Right		
○	○	**0**	Athlete is able to **effectively use arms** to assist with the test performance
○	○	**1**	Athlete is able to **use arms a little bit** to assist with test performance
○	○	**2**	Athlete **does not use arms** to assist with the test performance

13. 4 Bounds for Distance

Protocol / Description:

Participants started on a marked line and were instructed to cover the maximum possible distance in four consecutive, single-leg bounds from a standing start. The first bound was from their non-preferred leg, landing on their outstretched preferred leg. Using forward momentum to continue the movement, the second bound was conducted as they leapt from their preferred leg to their non-preferred leg. This pattern was repeated for a total of 4 bounds. Distance is measured from the starting line to the heel strike of the fourth bound (m).

Outcome: Distance covered with 4 bounds (m)

Source: Beckman, E.M. & Tweedy, S.M. (2009) Towards evidence-based classification in Paralympic athletics: evaluating the validity of activity limitation tests for use in classification of Paralympic running events. *British Journal of Sports Medicine*, 43, 1067-1072

Observation Categories

What of the next aspects of the athletes' performance have more impact on their performance?

A. Coordination:

- ◯ **0** Impaired coordination had **no impact** on the test outcome.
- ◯ **1** Impaired coordination had a **minor impact** on the test outcome.
- ◯ **2** Impaired coordination had a **major impact** on the test outcome.

B. Balance:

- ◯ **0** Impaired balance had **no impact** on the test outcome.
- ◯ **1** Impaired balance had a **minor impact** on the test outcome.
- ◯ **2** Impaired balance had a **major impact** on the test outcome.

C. Symmetry:

- ◯ **0** Impaired symmetry had **no impact** on the test outcome.
- ◯ **1** Impaired symmetry had a **minor impact** on the test outcome.
- ◯ **2** Impaired symmetry had a **major impact** on the test outcome.

D. Range of Movement:

- ◯ **0** Impaired range of movement had **no impact** on the test outcome.
- ◯ **1** Impaired range of movement had a **minor impact** on the test outcome.
- ◯ **2** Impaired range of movement had a **major impact** on the test outcome.

E. Arms impairment:

- ◯ **0** Athlete is able to **effectively use arms** to assist with the test performance.
- ◯ **1** Athlete is able to **use arms a little bit** to assist with test performance.
- ◯ **2** Athlete **does not use arms** to assist with the test performance.

14. 10m Speed Skip

Protocol / Description:

Markers were placed at 0, 10 and 20 m with pairs of infrared timing light gates positioned at the 10 and 20 m markers. Participants performed the skip—a hop–step– hop pattern—and were given an opportunity to practice until they could successfully complete the pattern over 10 m. Participants accelerated over the first 10 m so that they were at top speed when they reach the first light gate (10 m), and maintained top-speed as they moved through to the second gate (20 m).

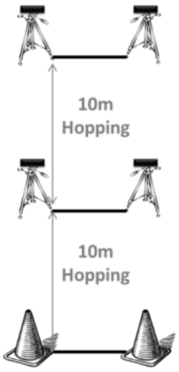

Outcome: Time (s) to move from 10 to 20 m was recorded.

Source: Beckman, E.M. & Tweedy, S.M. (2009) Towards evidence-based classification in Paralympic athletics: evaluating the validity of activity limitation tests for use in classification of Paralympic running events. *British Journal of Sports Medicine*, 43, 1067-1072

Observation Categories

What of the next aspects of the athletes' performance have more impact on their performance?

A. Coordination:

- ○ **0** Impaired coordination had **no impact** on the test outcome.
- ○ **1** Impaired coordination had a **minor impact** on the test outcome.
- ○ **2** Impaired coordination had a **major impact** on the test outcome.

B. Balance:

- ○ **0** Impaired balance had **no impact** on the test outcome.
- ○ **1** Impaired balance had a **minor impact** on the test outcome.
- ○ **2** Impaired balance had a **major impact** on the test outcome.

C. Symmetry:

- ○ **0** Impaired symmetry had **no impact** on the test outcome.
- ○ **1** Impaired symmetry had a **minor impact** on the test outcome.
- ○ **2** Impaired symmetry had a **major impact** on the test outcome.

D. Range of Movement:

- ○ **0** Impaired range of movement had **no impact** on the test outcome.
- ○ **1** Impaired range of movement had a **minor impact** on the test outcome.
- ○ **2** Impaired range of movement had a **major impact** on the test outcome.

E. Arms impairment:

- ○ **0** Athlete is able to **effectively use arms** to assist with the test performance.
- ○ **1** Athlete is able to **use arms a little bit** to assist with test performance.
- ○ **2** Athlete **does not use arms** to assist with the test performance.

15. Stop and Go Test

Protocol / Description:

The athlete stood without support behind the starting line, and started to run at the researchers signal. The athlete ran to a mat (10m) and stopped completely on the mat with both feet. After the first contact, the athlete remained on the mat for 2 seconds until a beep sounded. Immediately at the sound they ran again to the next mat (10m) and stopped again until the next beep, and then continued to the final mark at 10m from the second mat. Total distance = 30m.

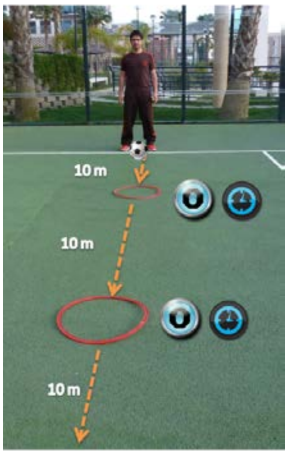

Outcome: time (seconds), measured with time gates to first mat (at 10m), second mat (at 20m), last gate (at 30m), total time (30m distance)

Source: Proposed by Reina, R. (2012) for Bicici, S., Tweedy, S. & Vanlandewijck, Y. (2012). Development of a test battery for improving classification reliability for ambulant athletes affected by hypertonia, ataxia or athetosis. KU Leuven: Unpublished Master Thesis.

Observation Categories

What of the next aspects of the athletes' performance have more impact on their performance?

A. Coordination:

- ◯ **0** Impaired coordination had **no impact** on the test outcome.
- ◯ **1** Impaired coordination had a **minor impact** on the test outcome.
- ◯ **2** Impaired coordination had a **major impact** on the test outcome.

B. Balance:

- ◯ **0** Impaired balance had **no impact** on the test outcome.
- ◯ **1** Impaired balance had a **minor impact** on the test outcome.
- ◯ **2** Impaired balance had a **major impact** on the test outcome.

C. Symmetry:

- ◯ **0** Impaired symmetry had **no impact** on the test outcome.
- ◯ **1** Impaired symmetry had a **minor impact** on the test outcome.
- ◯ **2** Impaired symmetry had a **major impact** on the test outcome.

D. Range of Movement:

- ◯ **0** Impaired range of movement had **no impact** on the test outcome.
- ◯ **1** Impaired range of movement had a **minor impact** on the test outcome.
- ◯ **2** Impaired range of movement had a **major impact** on the test outcome.

E. Arms impairment:

- ◯ **0** Athlete is able to **effectively use arms** to assist with the test performance.
- ◯ **1** Athlete is able to **use arms a little bit** to assist with test performance.
- ◯ **2** Athlete **does not use arms** to assist with the test performance.

16. 40m Sprint

Protocol / Description:

Athletes ran at maximum speed from a standing start to 40m. Timing light gates are positioned at 0, 10, 25 and 40 m

Outcome: time (s) to complete 10m, 25m and 40m.

Source: modified from Beckman, E.M. & Tweedy, S.M. (2009). Towards evidence-based classification in Paralympic athletics: evaluating the validity of activity limitation.

Observation Categories

What of the next aspects of the athletes' performance have more impact on their performance?

A. Coordination:

- ○ **0** Impaired coordination had **no impact** on the test outcome.
- ○ **1** Impaired coordination had a **minor impact** on the test outcome.
- ○ **2** Impaired coordination had a **major impact** on the test outcome.

B. Balance:

- ○ **0** Impaired balance had **no impact** on the test outcome.
- ○ **1** Impaired balance had a **minor impact** on the test outcome.
- ○ **2** Impaired balance had a **major impact** on the test outcome.

C. Symmetry:

- ○ **0** Impaired symmetry had **no impact** on the test outcome.
- ○ **1** Impaired symmetry had a **minor impact** on the test outcome.
- ○ **2** Impaired symmetry had a **major impact** on the test outcome.

D. Range of Movement:

- ○ **0** Impaired range of movement had **no impact** on the test outcome.
- ○ **1** Impaired range of movement had a **minor impact** on the test outcome.
- ○ **2** Impaired range of movement had a **major impact** on the test outcome.

E. Arms impairment:

- ○ **0** Athlete is able to **effectively use arms** to assist with the test performance.
- ○ **1** Athlete is able to **use arms a little bit** to assist with test performance.
- ○ **2** Athlete **does not use arms** to assist with the test performance.
